# AcrAB Efflux Pump Plays a Crucial Role in Bile Salts Resistance and Pathogenesis of *Klebsiella pneumoniae*

**DOI:** 10.3390/antibiotics13121146

**Published:** 2024-11-29

**Authors:** Rundong Shu, Ge Liu, Yunyu Xu, Bojun Liu, Zhi Huang, Hui Wang

**Affiliations:** 1 Sanya Institute of Nanjing Agricultural University, College of Life Sciences, Nanjing Agricultural University, Nanjing 210095, China; 2018116064@njau.edu.cn (R.S.); 2017116096@njau.edu.cn (G.L.); 2022116047@stu.njau.edu.cn (Y.X.); 10317107@njau.edu.cn (B.L.); 2 Zhengzhou Agricultural Science and Technology Research Institute, Zhengzhou 450015, China

**Keywords:** *Klebsiella pneumoniae*, bile salts, efflux pump, virulence, colonization, pathogenesis

## Abstract

Bile salts possess innate antibacterial properties and can cause significant damage to bacteria. To survive in the mammalian gut, *Klebsiella pneumoniae* has developed mechanisms to tolerate bile salts; however, the specific mechanisms remain unclear. Transposon library screening revealed that the efflux pump AcrAB is involved in bile salt resistance. *acrA* and *acrB* mutants exhibited high sensitivity not only to bile salts but also to SDS and various antibiotics, with a switch-loop, comprising residues G615, F616, A617, and G618, proving to be crucial in this process. A colonization defect of *acrA* and *acrB* mutants was demonstrated to be located in the mouse small intestine, where the bile salt concentration is higher compared to the large intestine. Additionally, both *acrA* and *acrB* mutants displayed reduced virulence in the *Galleria mellonella* model. In conclusion, our results suggest that the Resistance-Nodulation-Cell Division efflux pump serves as a critical determinant in the pathogenesis of *K. pneumoniae* through various aspects.

## 1. Introduction

Bile salts, representing the cationic counterparts of bile acids, manifest as amphipathic cholesterol derivatives. They are synthesized in the liver and are discharged into the gastrointestinal tract concomitant with food ingestion, attaining notable millimolar concentrations within the small intestine [1,2]. Beyond their well-documented role in lipid emulsification, bile salts are recognized for their potent antimicrobial properties [3]. Lorenzo observed the capacity of orally administered bile salts to curb bacterial overgrowth in cirrhotic rats [4], while Cremers illuminated the mechanism through which bile salts restrict bacterial proliferation, involving the widespread unfolding and aggregation of cytosolic proteins [5]. The cytotoxic effects of bile salts are intrinsically tied to their amphipathic nature. Their detergent-like properties facilitate the dissolution of bacterial cell membranes, inflict damage to DNA, induce conformational changes in proteins, and sequester iron and calcium ions [6,7]. Consequently, it is incumbent upon gut-colonizing bacteria to possess mechanisms that confer tolerance to bile salts. Several investigations have unveiled the diverse strategies employed by bacteria in confronting the challenges posed by bile stress. These strategies encompass the utilization of multidrug efflux pumps, exemplified by AcrAB-TolC and CemABC, which are presumed to be instrumental in resistance by expelling bile salts [8,9]. A suite of enzymatic reactions assumes an essential role in mitigating the adverse effects of bile salts. The bile salt hydrolase (*bsh*) enzymes catalyze taurine-conjugated bile acids into free bile acids, which can be further metabolized by other gut bacteria [10,11]. Furthermore, *Salmonella typhimurium* DNA adenine methylase mutants exhibit heightened susceptibility to bile salts, indicative of an impaired ability to repair DNA damage instigated by these compounds [7].

*Klebsiella pneumoniae*, an opportunistic pathogen, poses a substantial threat to human health due to its high virulence, causation of diverse diseases, and facile inter-host transmission. Two primary host niches for *K. pneumoniae* colonization are the upper respiratory tract and the gastrointestinal tract [12]. Additionally, *Klebsiella* is often isolated from bile or from infections associated with gallstones, the gallbladder, or bile ducts [13]. To establish successful infection, *K. pneumoniae* must overcome the toxic challenge of bile salts. Chen reported that loss of PgaC, a processive β-glycosyltransferase of the GT-2 family, affect the enhancement of *K. pneumoniae* CG43 biofilm in response to a bile salt mixture [14]. Tan observed that the inactivation of *wza* (capsule exportase) and *wzy* (capsule polymerase) in K1 hypervirulent *K. pneumoniae* SGH10 confers cell envelope defects in addition to capsule loss, making them susceptible to bile salts and detergent stress [15]. However, the mechanisms of bile salt resistance regulation and its association with antibiotic resistance in *K. pneumoniae* remain unclear. In this study, we employed transposon random mutagenesis to screen for genes responsible for bile salts tolerance, aiming to elucidate the potential mechanisms of bile salts in *K. pneumoniae*.

## 2. Results

### 2.1. Tolerance of Clinical K. pneumoniae Isolates to Bile Salts

**Figure 1:**
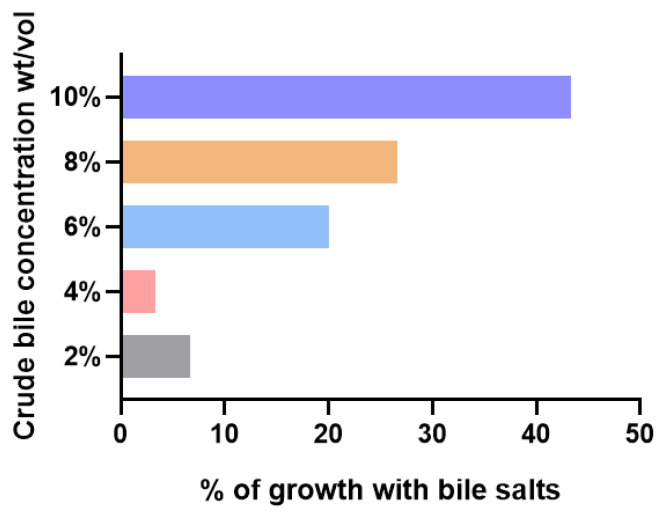
Bile salt tolerance distribution among *K. pneumoniae* clinical strains. Overnight culture of each *K. pneumoniae* clinical strain was transferred to fresh LB broth containing different concentrations of crude bile (2, 4, 6, 8 and 10%). Growth conditions were assessed based on optical density after 16 h of incubation aerobically.

Bile salt concentrations in the mammalian small intestine generally range from 0.2% to 2% (*w*/*v*) [8]. To investigate the effect of bile on *K. pnuemoniae* growth, we first compared the growth of 30 *K. pnuemoniae* clinical strains that were isolated from various organs (Table S1) in LB medium supplemented with up to 10% crude bile (Figure 1). We found that 93.3% of strains tested were resistant to 2% crude bile, with 46.7% of the strains showing tolerance in the presence of 10% crude bile. These results suggest a common adaptation of *K. pnuemoniae* clinical strains to bile salts, independent of their isolation source.

### 2.2. AcrAB Is Involved in Resistance to Bile Salts in K. pneumoniae

**Figure 2:**
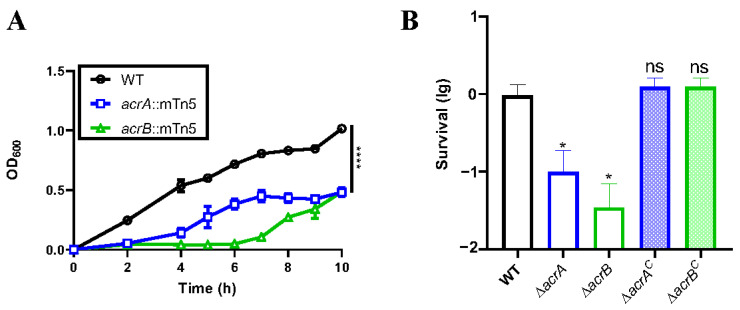
The growth of Δ*acrA* and Δ*acrB* mutant with crude bile. (**A**) Growth curve of strains in the presence of crude bile. cultures of the wild-type and candidate mutants were initiated by inoculating them at a 1:100 ratio into fresh LB medium in the presence of 0.5% crude bile. These cultures were then incubated with agitation at 37 °C, and the OD_600_ was recorded at the specified time points. (**B**) Bile salt killing assay. Stationary cultures of wild-type, mutants, and complemented strains were diluted into either saline or saline containing 0.5% crude bile and were subsequently incubated for 1 h. The viable cell counts were determined through serial dilution and plating. Survival rates were calculated by normalizing CFU to the bile salt-treated group. The data represent the means ± SDs of results from three independent experiments. Significance levels are indicated as follows: *, *p* < 0.05; ****, *p* < 0.0001; ns, no statistical significance (Student *t*-test).

To elucidate the mechanisms underpinning resistance to bile salts, we constructed a transposon library of approximately 10,000 random transposon insertion mutants derived from *K. pneumoniae* A2312 using the mTn5 transposon, enabling the identification of bile-sensitive mutants. Within this screen, we identified four mutants that displayed pronounced hypersensitivity to 0.5% crude bile (Figure S1A). Subsequent genetic analyses revealed that four genes (*acrA*, *acrB nplD*, *ruvB*) were involved in transposon insertions (Figure S1B). Finally, we focused on two candidate genes, *acrA* and *acrB*. Notably, the *acrA*::mTn5 mutant exhibited impaired growth in the presence of 0.5% crude bile, while the *acrB*::mTn5 mutant displayed even more sluggish growth (Figure 2A). Growth in Luria–Bertani medium for both the wild-type strain and the mutants was virtually indistinguishable, as confirmed in Figure S2, affirming that the heightened susceptibility of the *acrA* and *acrB* mutants to bile salts was unrelated to a growth deficiency.

AcrAB-TolC, part of the Resistance–Nodulation–Cell Division (RND) family, is composed of three key components: AcrB, AcrA, and TolC [16]. AcrB, situated in the inner membrane, assumes the roles of energy transduction and substrate selection within the pump assembly. AcrA functions as an adapter element, mediating the connection between the inner membrane pump and the outer membrane channel TolC. TolC forms a conduit traversing the outer membrane, offering an efflux pathway for substances translocated from the inner membrane and the periplasmic space. The combined action of these three components is essential for bacterial survival and the execution of multidrug resistance mechanisms [8]. We further constructed mutants for *acrA* and *acrB*, employing the pVIK112 vector, hereafter designated ∆*acrA* and ∆*acrB*, respectively [17]. To corroborate these gene function, a killing assay was conducted, which confirmed a comparable outcome, as depicted in Figure 2B. The survival rate of ∆*acrA* mutant in LB medium containing 0.5% crude bile exhibited a significant 10-fold reduction, while ∆*acrB* mutant experienced a 25-fold decrease in survival. To restore the functionality of the mutant, we incorporated gene fragments of *acrA* or *acrB* into the pSRKGm vector and subsequently introduced the recombinant plasmids into the corresponding mutants, leading to the creation of complementary strains, denoted ∆*acrA*^C^ and ∆*acrB*^C^ [18]. Figure 2B demonstrates that ∆*acrA*^C^ and ∆*acrB*^C^ strains reinstated the phenotype observed in the wild-type strain under conditions of crude bile exposure.

Furthermore, we conducted a comparative assessment of the survival rates between the wild-type strain and the mutants when exposed to 0.5% crude bile at distinct growth phases (the lag phase, mid-log phase, and stationary phase) (Figure S3). Both Δ*acrA* and Δ*acrB* mutants displayed heightened susceptibility to crude bile. When contrasted with the wild-type strain, the survival rates of Δ*acrA* and Δ*acrB* were substantially diminished by approximately 10^3^-fold during the lag phase, 10^2^-fold during the mid-log phase, and 10-fold during the stationary phase. These findings substantiate the critical role of AcrAB in protecting *K. pneumoniae* against the deleterious effects of bile salts across all growth phases.

### 2.3. AcrAB Mutants Are More Susceptible to Antimicrobial Agents

**Figure 3:**
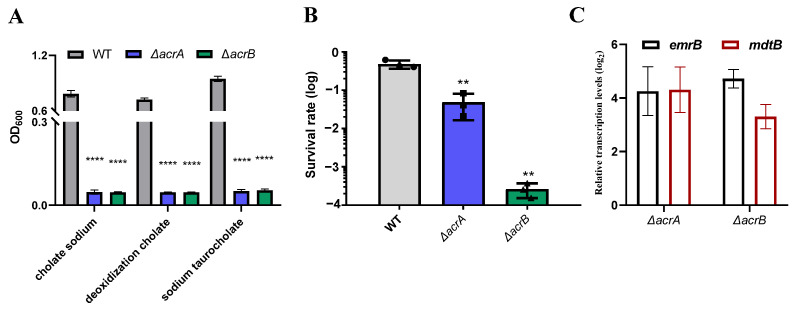
Effects of antimicrobial agents on multidrug efflux pump AcrAB mutants. (**A**) Growth of wild-type, ∆*acrA*, and ∆*acrB* mutants in the presence of different bile salts. Overnight cultures of wild-type and the mutants were diluted 1:100 into fresh LB medium with or without 0.5% sodium cholate, deoxidized cholate, or sodium taurocholate, respectively, and incubated with shaking at 37 °C. OD_600_ was measured after 10 h. (**B**) SDS killing assay. Stationary cultures of wild-type and the mutants were diluted into either saline or saline containing 0.5% SDS. After a 1 h incubation, viable cells were enumerated. The survival rate was calculated by normalizing the CFU to the group treated with SDS. (**C**) The effects of AcrAB on *emrB* or *mdtB* gene transcription. RNA was extracted from mid-logarithmic growth phase cells of both the wild-type and mutant strains. The extracted RNA was then subjected to RT-qPCR analysis to assess the expression levels of the *emrB* and *mdtB* genes. qRT-PCR was performed and the results were normalized against 16S rRNA as the internal reference. The data are presented as means ± SDs based on the results of three independent experiments. Statistical significance is indicated as follows: **, *p* < 0.01; ****, *p* < 0.0001 (Student *t*-test).

Crude bile represents a heterogeneous mixture primarily composed of cholate and deoxycholate [19]. To elucidate the substrate specificity of the AcrAB efflux pump, we conducted an examination of the growth of Δ*acrA* and Δ*acrB* mutants in the presence of three distinct bile salts: sodium cholate, sodium taurocholate, and deoxycholate. Following a 12 h incubation, the OD_600_ for the wild-type strain reached 1.0, whereas the mutants exhibited barely increased (Figure 3A). This observation suggests that both the *acrA* and *acrB* genes play a role in conferring resistance to various bile salt types, devoid of strict specificity.

Efflux pumps play a vital role in the export of various antimicrobial agents, encompassing both antibiotics and non-antibiotics. Nikaido reported that the AcrAB deletion mutant in *Salmonella typhimurium* SH7616 exhibited heightened sensitivity to detergent sodium dodecyl sulfate (SDS) and several antibiotics [20]. We further selected SDS as a non-antibiotic compound to investigate the functional role of AcrAB in *K. pneumoniae* by conducting a killing assay. In LB medium containing 0.5% SDS, the survival rate for the wild-type strain reached 4.83 × 10^−1^, whereas that of the ∆*acrA* strain and ∆*acrB* strain significantly plummeted to 4.83 × 10^−2^ and 2.62 × 10^−4^, respectively (Figure 3B). These results incontrovertibly establish that the AcrAB pumps confer SDS resistance to *K. pneumoniae*.

Regarding antibiotic tolerance, we determined the antimicrobial susceptibility by dilution and plate counting for five distinct antibiotic classes in the Δ*acrA* and Δ*acrB* mutants, as summarized in Table S2. Our findings revealed that these mutant strains exhibited sensitivity to quinolones, polyketides, and rifamycins, while displaying no discernible growth defects in the presence of aminoglycosides or β-lactams. Specifically, the Δ*acrA* mutants displayed approximately a 5-fold reduction in minimum inhibitory concentration (MIC) for chloramphenicol in comparison to the wild-type, as well as a 2-fold reduction in MIC for tetracycline and rifampicin. On the other hand, the Δ*acrB* mutants exhibited approximately a 2-fold reduction in MIC for chloramphenicol, a 3-fold reduction in MIC for rifampicin, and a slight change in tetracycline susceptibility. The observed susceptibility pattern for chloramphenicol, tetracycline, and rifampicin aligns with reports from studies involving *S. typhimurium* SH7616 [20]. Complementary strains Δ*acrA*^C^ and Δ*acrB*^C^ fully reinstated tetracycline tolerance and partially restored resistance to chloramphenicol and rifampicin. This suggests that AcrAB plays a contributory role in resistance against select antibiotic classes.

Most bacteria possess multiple families of efflux pumps that contribute to intrinsic or acquired resistance against a diverse spectrum of antibiotics. Notable among these are the RND superfamily, which includes AcrAB-TolC, AcrD, MdtABC, and KexD, as well as the Major Facilitator Superfamily (MFS), exemplified by EmrAB. Lomovskaya demonstrated the involvement of EmrAB in *Escherichia coli* in the expulsion of bile salts and nalidixic acid from the bacterial cell [21]. Fei’s research on *K. pneumoniae* KPN142 unveiled the role of MdtABC in excluding β-lactamase antibiotics, in addition to AcrAB [22]. To investigate the potential synergy between these efflux pumps, we quantified the expression levels of *emrB* and *mdtB* in the Δ*acrA* and Δ*acrB* mutants using quantitative real-time PCR (RT-qPCR). Strikingly, we observed a substantial increase in the expression of *emrB* and *mdtB*, approximately 22-fold in the Δ*acrA* mutant compared to the wild-type. Likewise, the Δ*acrB* mutant exhibited a 25-fold increase in *emrB* expression and a 10-fold increase in *mdtB* expression, as depicted in Figure 3C. These findings determine the enhanced activity of the efflux pumps MdtABC and EmrAB in the absence of AcrAB, suggesting an intricate interplay among these systems.

### 2.4. Switch-Loop Is the Key Function Site of AcrB Protein

**Figure 4:**
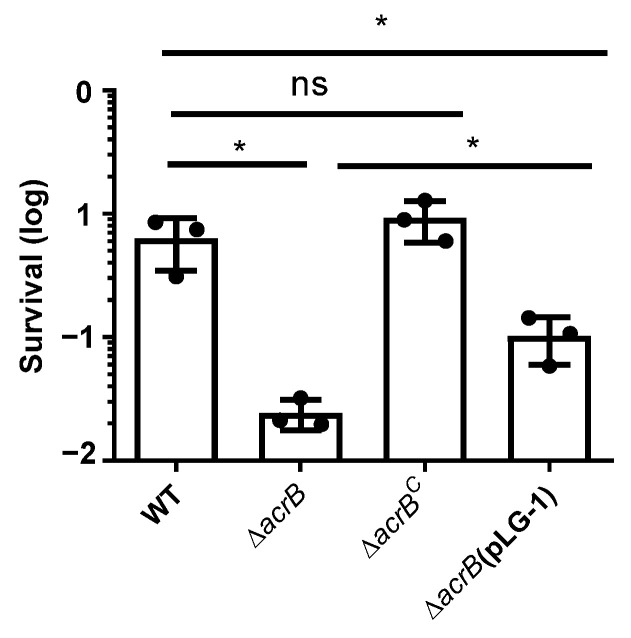
Influence of the switch-loop on bile salt tolerance in *K. pneumoniae*. Cultures in stationary phase of wild-type, Δ*acrB* mutant, Δ*acrB*^C^, and partial complemented strain Δ*acrB*(pLG-1) were diluted into both saline and saline containing 0.5% crude bile. After one-hour incubation, viable cells were counted. The survival rate was calculated by normalizing the CFU to the crude bile-treated group. The data represent the means ± SDs of three independent experiments. *, *p* < 0.05; ns, no statistical significance (Student *t*-test).

Our findings indicate that the absence of the *acrB* gene exerts a more pronounced impact on antimicrobial agent tolerance compared to the absence of the *acrA* gene. AcrB, an important component of the pump assembly, serves as both the energy transducer and the substrate transporter, employing an allosterically coupled rotational mechanism in which each monomer sequentially adopts one of three distinct conformations [16]. Notably, a key switch-loop, consisting of 11 amino acids (residues 613–623), plays a critical role in separating two binding pockets within the crystal structure of *E. coli* AcrB [23]. This structural element is of paramount importance in expelling antimicrobial compounds. Jamshidi confirmed that the *K. pneumoniae* AcrB transporter exhibits a high degree of similarity, with a sequence identity of 91.6%, to the crystal structure of *E. coli* AcrB [24]. This indicates that the switch-loop structure in *K. pneumoniae* AcrB may play a similarly critical role. To investigate this further, we constructed a *K. pneumoniae* A2312 *acrB* deletion variant, specifically lacking residues 615–618, hereafter designated as Δ*acrB*(pLG-1). We conducted an examination of bile salt sensitivity, as illustrated in Figure 4. Our results reveal that the survival rate of the switch-loop mutant Δ*acrB*(pLG-1) (1.02 × 10^−1^) falls between that of the wild-type (6.35 × 10^−1^) and the Δ*acrB* mutant (2.43 × 10^−2^), while the complementary strain Δ*acrB*^C^ (9.24 × 10^−1^) can fully restore the wild-type level of resistance.

The SDS killing assay produced analogous results: the survival rate of Δ*acrB*(pLG-1) ranged from 10^−3^ to 10^−2^, which was lower than that of both the wild-type and Δ*acrB*^C^ strains (both ranging from 10^−1^ to 1), but higher than that of the Δ*acrB* mutant (ranging from 10^−5^ to 10^−4^) (Figure S4). In terms of antibiotic tolerance, the partially complemented Δ*acrB* mutants (Δ*acrB*(pLG-1)) exhibited a marginal increase in rifampicin tolerance, albeit without a discernible impact on chloramphenicol and tetracycline resistance (Table S2). These findings underscore the crucial role of the switch-loop in the functionality of AcrB.

### 2.5. AcrAB Affects the Small Intestinal Colonization of K. pneumoniae

**Figure 5:**
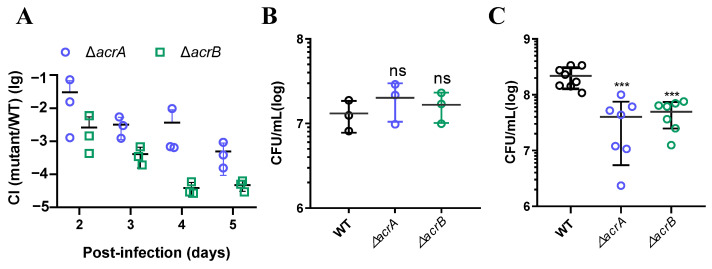
The effect of multidrug efflux pump AcrAB in intestinal colonization. (**A**) Adult mouse competition assays. A total of 10^8^ cells of the wild-type and Δ*acrA* or Δ*acrB* mutants were mixed in a 1:1 ratio and intragastrical administered to mice. Fecal pellets were collected from each mouse at the indicated time points and plated onto selective agar plates. The competitive index [28] was calculated as the ratio of mutant to wild-type colonies normalized to the input ratio. The horizontal line represents the mean CI of 3 mice. (**B**,**C**) Colonization of *K. pneumoniae* using an M-E-A-T model. Intestinal tissues were collected from three mice, each measuring approximately 2.0 cm in length. Subsequently, 200 µL of *K. pneumoniae* cells at a concentration of approximately 2 × 10^7^ CFU were added to the inside-out tissues in a 30 mm Petri dish. After a 4 h incubation, the intestinal tissues were homogenized in 3 mL of PBS. Bacteria were enumerated by serial dilution and plated onto selective agar plates. Bacterial CFU in the large intestine (**B**) and bacterial CFU in the small intestine (**C**) are shown. ***, *p* < 0.001; ns, no statistical significance (Student *t*-test).

Various host and commensal microbial signals regulate pathogen gene expression in vivo. Hsiao demonstrated that a *B. obeum*-produced AI-2 autoinducer can downregulate the expression of genes related to toxin-coregulated pili biosynthesis during *Vibrio cholerae* infection [25]. Given the intricate nature of the in vivo environment, we sought to explore the impact of AcrAB in *K. pneumoniae* pathogenesis. To this end, we conducted a competitive colonization experiment using an adult mouse model [26]. Equal volumes of 10^8^ cells from the wild-type strain and Δ*acrA* or Δ*acrB* mutant strains were mixed and administered to the mice intragastrically. Fecal pellets were collected daily to determine the ratio of the mutant strain to the wild-type strain. As depicted in Figure 5A, both the Δ*acrA* and Δ*acrB* mutants were consistently outcompeted by the wild-type strain. The competitive index (CI) of the Δ*acrA* mutant exhibited a continual decline, starting at 2.99 × 10^−2^ on the 2nd day post-infection, and eventually stabilized at 4.92 × 10^−4^ by the 5th day post-inoculation. The Δ*acrB* mutants exhibited a weaker ability to colonize compared to that of the Δ*acrA* mutant. Specifically, the period from the 2nd day to the 5th day saw a downward trend in the CI of Δ*acrB* mutants from 2.6 × 10^−3^ to 4.73 × 10^−5^, which was significantly lower than that of the Δ*acrA* mutant. This observation implies that the absence of the *acrB* gene exerts a more severe influence on *K. pneumoniae* maintenance in the intestine than the absence of the *acrA* gene. The gut is recognized as a mini-ecosystem housing a variety of complex environmental factors. Prouty reported that bile salts exhibit a steep gradient in concentration within the gut, with high levels in the small intestine and very low levels in the large intestine [9]. To further understand the role of the *acrAB* genes in colonization in different parts of the intestine, we employed a murine ex vivo anaerobic tissue (M-E-A-T) model to quantitatively assess their effect [27]. Our results indicate that both the Δ*acrA* and Δ*acrB* mutants colonized the large intestine as effectively as the wild-type (Figure 5B). However, in the small intestine, the CFU of the Δ*acrA* and Δ*acrB* strains was significantly reduced by 16-fold and 8-fold, respectively (Figure 5C). This suggests that the intestinal colonization defect of Δ*acrA* and Δ*acrB* may result from their sensitivity to bile salts, which are abundant in mammalian intestinal tracts and continuously recycled in the body through an efficient enterohepatic circulation.

### 2.6. The Virulence of ΔacrA and ΔacrB Mutants Were Reduced

To assess whether the AcrAB efflux pump impacts the virulence of *K. pneumoniae*, we employed a *Galleria mellonella* model, a straightforward system for assessing bacterial virulence [29]. Larvae were subjected to infection with varying concentrations of *K. pneumoniae* A2312 cells, ranging from 10^3^ to 10^6^ CFU, and their survival rates were monitored over a 48 h period (Figure S5A). The results indicated that mortality rates increased with higher doses of *K. pneumoniae* A2312. Larvae infected with 10^3^ CFU *K. pneumoniae* cells exhibited a mild effect, with a 100% survival rate within 24 h. Conversely, larvae infected with a higher dose (10^5^ CFU) of *K. pneumoniae* cells displayed a significant reduction in survival rates over time, with a 40% final survival rate at 24 h. In the case of larvae infected with a 10^6^ CFU inoculum, there was a substantial drop in survival rates, with only 40% surviving at 12 h and a further reduction to 10% at 24 h post-infection. This observation indicates a dose-dependent lethality in the larvae.

**Figure 6:**
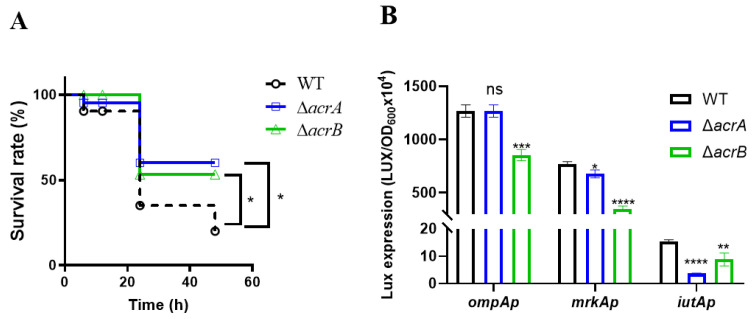
The effects of AcrAB on the virulence of *K. pneumoniae*. (**A**) Survival of *G. mellonella* larvae after injection with suspensions of wild-type or mutants. Impact of AcrAB on the survival rate of *K. pneumoniae* in *G. mellonella* larvae. Larvae were injected with 10 µL of PBS containing a lethal dose of approximately 10^7^ CFU of *K. pneumoniae*. Following the injection, the larvae were incubated at 37 °C in the dark, and the survival rate was recorded at specified time points (*n* = 20 larvae per group). *, *p* < 0.05 (Gehan–Breslow–Wilcoxon test). (**B**) The effect of AcrAB on the expression of the virulence genes of *K. pneumoniae*. Overnight cultures of the wild-type, Δ*acrA*, and Δ*acrB* mutants, each carrying P*_ompA_*-*luxCDABE*, P*_mrkA_*-*luxCDABE*, or P*_iutA_*-*luxCDABE* transcriptional fusion plasmids, were diluted 1:100 into LB broth. The cultures were incubated with shaking at 37 °C, and luminescence was measured at the stationary phase and subsequently normalized to the corresponding OD_600_. Data represents the means ± SDs of three independent experiments. *, *p* < 0.05, **, *p* < 0.01, ***, *p* < 0.001, ****, *p* < 0.0001, ns, no significance (Student *t*-test).

For further evaluation, a dose of 10^5^ CFU was selected as a suitable lethal dose to assess the virulence of the wild-type, Δ*acrA*, and Δ*acrB* mutants. As depicted in Figure 6A, larvae infected with the wild-type, Δ*acrA*, and Δ*acrB* mutants exhibited comparable survival rates within the initial 12 h. However, beyond this point, larvae infected with the wild-type exhibited a further gradual decline, with a survival rate reduced by approximately 60% at 24 h and 20% at 48 h. In contrast, larvae infected with the Δ*acrA* and Δ*acrB* mutants displayed a notably higher survival rate, reaching 60% and 55% at 24 h of infection, respectively, and maintaining this level through 48 h. To gain further insights, we retrieved hemolymph from the infected larvae at the indicated time points and quantified the bacterial load (Figure S5B). An equivalent bacterial count was retrieved from both wild-type and mutant-infected larvae, implying that the impairment of AcrAB may diminish the virulence of *K. pneumoniae* in vivo.

*K. pneumoniae* boasts an array of well-characterized virulence determinants. For instance, OmpA, a major component of outer membrane proteins, plays an essential role in mediating bacterial biofilm formation, eukaryotic cell infection, antibiotic resistance, and immunomodulation [30], MrkA encodes the major fimbriae subunit of Type 3 fimbriae [31], while IutA serves as a siderophore aerobactin transporter [32]. We further conducted a comparative assessment of the expression levels of *ompA*, *mrkA*, and *iutA* in the wild-type and mutant strains. This analysis was facilitated through the construction of promoter-*luxCDABE* transcriptional fusion plasmids [33]. In comparison to the wild-type, we observed varying degrees of reduction in the expression of *mrkA*, *ompA*, and *iutA* in different mutants, particularly at the stationary phase (Figure 6B). Specifically, the expression of the *ompA*, *mrkA*, and *iutA* genes was only slightly affected in the Δ*acrB* mutant. However, the Δ*acrA* mutant exhibited a noticeable reduction in the expression of the *iutA* gene. This suggests that this decrease in gene expression could be associated with the observed reduction in virulence.

## 3. Discussion

As one of the most innately bactericidal compounds present in humans, bile salts pose a challenge that many pathogens counteract through various resistance strategies [8]. For instance, Begley demonstrated *Listeria monocytogenes* L028 could grow on agar plates containing 15% oxgall, 15% bovine bile, or 2% porcine bile [34]. Similarly, Velkinburgh determined that the MIC of oxgall for stationary phase *S. typhimurium* and *Salmonella typhi* was 18% and 12%, respectively [35]. Remarkably, even under extremely high bile concentrations (exceeding 60%), *Salmonellae* can colonize the gallbladder [36]. The concentration of bile salts in the upper part of the human gastrointestinal tract typically ranges from 4 to 16 mM (approximately 2–6 mg/mL), a range influenced by factors such as the time of day, diet, and individual variations [37]. Ridlon reported a gradient in bacterial colonization in the small intestine, with bacterial densities increasing from around 10^3^ bacterial/mL in the duodenum to 10^4^ bacterial/mL in the jejunum, and reaching 10^6^ to 10^8^ bacterial/mL in the ileum [2]. Our growth test found that most clinically isolated *K. pneumoniae* strains survived in the presence of 10% crude bile (Figure 1). This observation underscores the significance of bile salt tolerance for *K. pneumoniae* in its survival and subsequent potential for infection.

The molecular mechanisms that bacteria employ to sense and counteract the effects of bile salts are notably intricate [8]. Lipopolysaccharide [38], multidrug efflux pumps [21,22,38], the two-component system PhoPQ [35], and porins [39] all play roles in conferring resistance to bile salts in Gram-negative bacteria. This study comprehensively screened for bile salt-sensitive mutants in *K. pneumoniae* A2312 using transposon mutagenesis library. Our findings align with observations in other bacteria such as *Salmonella* [38], *Shigella* [40], and *E. coli* [39], highlighting the role of the RND efflux transporter AcrAB in resisting bile salts, without specificity for bile salt types (Figure 2A,B and Figure 3A) [24].

AcrAB is characterized for its role in effluxing a wide array of substrates in *E. coli* [24], encompassing indole, short-chain fatty acids, detergents, and antibiotics [16]. This efflux activity provides bacteria with a means to survive in hostile environments. Our results were consistent with these previous reports, as we observed an increased susceptibility to SDS (Figure 3B), quinolones, polyketones, and rifamycins in the Δ*acrA* and Δ*acrB* mutants. Notably, the tolerance of these mutants to aminoglycosides and β-lactams remained unaltered in comparison to the wild-type (Table S2). *K. pneumoniae* possesses a range of efflux pumps, including AcrAB, MdtABC, and EmrAB [8]. Subsequent measurements revealed a dysregulation of *emrB* and *mdtB* following disruption of the AcrAB efflux pumps, which could potentially account for the observed unaltered sensitivity to aminoglycosides and β-lactams (Figure 3C).

AcrA serves as the adapter component, facilitating the connection between the inner membrane pump and the outer membrane channel TolC, while AcrB is situated in the inner membrane, where it functions as the energy transducer and substrate selector for the pump complex [16]. Results indicate that the absence of *acrB* has a more pronounced impact than *acrA* on resistance to antimicrobial compounds and the ability of *K. pneumoniae* to persist in the intestinal environment, and the disrupting of the switch-loop of AcrB in *K. pneumoniae* A2312 partially impairs its function (Figure 4).

Previous research by Padilla identified AcrB transporters as a novel virulence factor, and the knocking out of *acrB* gene significantly reduced the ability of *K. pneumoniae* 52,145 to cause pneumonia in mice [41]. In our study, both Δ*acrA* and Δ*acrB* mutants similarly exhibited a colonization defect in an adult mouse model (Figure 5A). Further validation through a murine ex vivo anaerobic tissue model pinpointed the precise location of this colonization defect in the small intestine (Figure 5B,C), where bile salt concentrations are notably higher than in the large intestine [9]. Virulence is another key factor in evaluating pathogenicity. In a *Caenorhabditis elegans* model, Bialek demonstrated that the deletion of the *acrB* gene results in a reduced virulence in the KPBj1E + Δ*acrB* strain [42]. Our finding reveals a significant decrease in the expression of virulence gene *mrkA*, *ompA*, and *iutA* in Δ*acrA* and Δ*acrB* mutants (Figure 6B). Murphy reported that the presence of type 3 fimbriae in *K. pneumoniae* was associated with reduced colonization and persistence in a mouse model [31]. Skerniskyte observed that certain bacteria with functional OmpA proteins were capable of eliciting a heightened proinflammatory response [28]. Dozois identified that avian pathogenic *E. coli* χ7122 induces *iutA* expression during chicken infection through the selective capture of transcribed sequences [32]. Efflux pumps are also involved in the regulation of virulence factors. Vaillancourt found that *P. aeruginosa mexR* and *mexEF* efflux pump variants exhibit increased virulence during acute murine lung infection [43]. Mateus confirmed that the RDN efflux system contributes to the virulence of *Aliarcobacter butzleri* [44]. These could be the reasons why the absence of the *acrA* or *acrB* gene reduces the lethality of *K. pneumoniae* in the *G. mellonella* model (Figure 6A).

Due to the crucial role in antimicrobial resistance and pathogenesis, AcrAB presents substantial potential as a target for therapeutic intervention against *K. pneumoniae*. Developing ligands that specifically bind to AcrAB proteins could enhance the antibacterial effects of quinolones and other antibiotics and reduce the virulence and colonization ability of *K. pneumoniae*, which address the dual challenges of antibiotic resistance and bacterial virulence more effectively.

## 4. Materials and Methods

### 4.1. Ethics Statement

All animal studies were conducted in strict accordance with the approved animal protocols, as sanctioned by the Ethical Committee of Animal Experiments at Nanjing Agricultural University, under permit SYXK [Su] 2017-0007.

### 4.2. Bacterial Strains, Plasmids, and Growth

All *K. pneumoniae* strains used in this study were obtained from the Chinese Center for Disease Control and Prevention (China CDC). Subsequent studies were conducted using a clinical ST11-K47 isolate A2312 as the wild-type strain [45]. All *E. coli* and *K. pneumoniae* strains were cultivated in LB medium or on LB agar, supplemented with the appropriate antibiotics, at a temperature of 37 °C, unless otherwise specified.

To construct the insertion mutants, an internal fragment of the *acrA*/*acrB* was amplified by PCR and cloned into plasmid pVIK112 [17]. The resulting plasmids were introduced into *K. pneumoniae* by conjugation. Single homologous recombination mutants were selected, resulting in the insertion of pVIK112 into the target gene, flanked by two truncated copies of the target gene. Complemented strains were constructed by cloning the *acrA/acrB* coding sequences into pSRKGm containing the *lac* promoter [18], and these plasmids were introduced into the *acrA/acrB* mutant strain to create the complemented and overexpressed strains (hereafter referred to as *acrA^C^* and *acrB^C^*). To construct the *acrB* mutant variant lacking amino acid residues 615–618, the truncated *acrB* gene sequence, with the region between 1843 bp and 1854 bp excised, was generated using overlapping PCR. The resulting fragment was ligated into the plasmid pSRKGm to create the recombinant plasmid pLG-1, which was then transferred into the *acrB* mutant strain via conjugation. Transcriptional fusion reporters were established by cloning the promoter sequences of the target genes into a plasmid containing a promoterless *luxCDABE* reporter [33].

### 4.3. Bile Salt Susceptibility Tests

Bile salt susceptibility was measured by monitoring the culture OD_600_ in various assays. Briefly, overnight cultures of each tested strain were inoculated 1:100 into LB medium containing appropriate bile salts and antibiotics, then incubated at 37 °C with shaking at 180 rpm. For the assay of clinical isolates, crude bile concentrations of 2%, 4%, 6%, 8%, and 10% were used. During mutant screening and further property assays, 0.5% crude bile was applied. To evaluate the effects of different bile salts on strain growth, 0.5% sodium cholate, deoxidized cholate, or sodium taurocholate (all from Sigma Chemical Co., St Louis, MO, USA) was added.

### 4.4. Bile Salt Sensitive Mutant Screening

For the creation of the transposon library, the conjugation process involved the utilization of *E. coli* BW20676(pRL27) as the donor strain and *K. pneumoniae* A2312 as the recipient strain [46]. After conjugation, transconjugants were separately streaked onto LB plates with or without 0.5% crude bile. Those mutants displaying heightened sensitivity to crude bile were subsequently isolated and earmarked for more comprehensive analysis. To pinpoint the precise insertion sites of the transposon, we conducted Arbitrary PCR and employed DNA sequencing [47].

### 4.5. Antibacterial Agent Killing Assay

The experimental procedure involved the initial preparation of overnight cultures for each test strain, which were subsequently diluted 1:100 in LB broth and cultivated with aeration. These cultures were then, at various growth phases, subjected to a 1:10 transfer into a solution containing 0.8% NaCl supplemented with 0.5% bile salts or 0.5% SDS. Following aerobic incubation for 1 h, the survival rate (%) was determined by enumerating viable cells through serial dilution in 0.8% NaCl and plating on LB agar. This entire process was repeated in triplicate for statistical rigor.

### 4.6. Minimum Inhibitory Concentration (MIC) Assay

Overnight cultures were subjected to a 1% transfer into fresh LB medium and allowed to reach the stationary growth phase. At this point, samples were withdrawn, and viable cell counts were determined by employing serial dilution techniques, followed by plating on LB agar plates containing varying concentrations of antibiotics, namely ampicillin, chloramphenicol, tetracycline, rifampicin, and streptomycin. The entire process was replicated three times to ensure the reliability of the results.

### 4.7. RT-qPCRs

We employed RT-qPCR to quantify the transcription levels of the target genes. Total RNA extraction was carried out using the TRIzol method [48] and subsequently reverse transcribed into cDNA using the cDNA Synthesis kit (Vazyme Biotech, Nanjing, China). The actual qPCR was conducted on a 7500 Plus real-time PCR system (Applied Biosystems, Foster City, CA, USA) in accordance with the SYBR green assay protocol (Vazyme Biotech, Nanjing, China). The reference gene utilized in this analysis was 16S rRNA [49]. To determine gene expression levels, the 2^−ΔΔCT^ method was applied [50]. Each group consisted of three independent replicates for statistical robustness.

### 4.8. Adult Mouse Colonization Assay

We conducted an adult mouse model experiment as previously outlined [26], with some adaptations. Six-week-old CD-1 mice were subjected to drinking water that included 0.5% (*w*/*v*) streptomycin and 0.5% (*w*/*v*) aspartame for the entire study duration. A day following the streptomycin treatment, a blend of WT and Δ*acrA* or Δ*acrB* mutant cultures, maintaining a 1:1 ratio and approximately 10^8^ CFU, was orally administered to the mice. Fecal pellets were gathered at specified time intervals, and the colonized bacteria were quantified through serial dilution and plated onto selective LB agar plates containing 100 µg/mL streptomycin (for both wild-type and mutant strain) or 50 µg/mL kanamycin (for mutant strain). After overnight incubation, the competitive index was computed as the ratio of recovered mutant colonies to wild-type colonies divided by the initial ratio of mutant to wild-type strain.

### 4.9. Murine Ex Vivo Anaerobic Tissue (M-E-A-T) Model

Intestinal tissues obtained from six-week-old CD-1 mice were dissected into approximately 2.0 cm-long fragments and arranged in dishes containing LB medium. Subsequently, 2 × 10^7^ CFU of *K. pneumoniae* cells were applied to the exposed intestinal tissues, and the dishes were maintained at 37 °C in an anaerobic chamber (O^2−^) [27]. After a 4 h incubation period, the intestinal tissues were homogenized. Bacteria were quantified through serial dilution and plating on selective LB agar plates containing 100 µg/mL streptomycin (for both wild-type and mutant strain) or 50 µg/mL kanamycin (for mutant strain).

### 4.10. Infection of G. mellonella Larvae

We employed *Galleria mellonella* larvae to evaluate the pathogenicity of *K. pneumoniae*, as previously described [29], with slight modifications. To determine a lethal dose of *K. pneumoniae* A2312, the last right proleg of each larva was surface disinfected with 70% ethanol. Subsequently, 10 μL of a series of 10-fold serial dilutions, ranging from 10^5^ to 10^8^ CFU, containing the wild-type strain resuspended in PBS buffer was injected using a Hamilton syringe equipped with a 50-gauge needle. To assess the impact of AcrAB on the survival rate of *K. pneumoniae* in *G. mellonella*, larvae were infected with 10 μL (10^5^ CFU) of WT, ∆*acrA*, or ∆*acrB* inoculum. The larvae were then incubated at 37 °C in the dark and monitored every 6 h for survival. Larvae that did not respond to physical stimuli were recorded as deceased (*n* = 20 larvae per group). To examine the ability of *K. pneumoniae* to persist in the hemolymph of *G. mellonella*, larvae were injected with 10 μL of bacterial suspensions in PBS at a concentration of 10^7^ CFU/mL. Hemolymph samples were collected at different time points, serially diluted, and plated for quantification (*n* = 3 larvae per group).

### 4.11. Measuring Transcriptional Expression Using Lux Reporters

Overnight cultures were diluted 1:100 in fresh LB broth and allowed to grow until they reached the stationary phase. Luminescence was measured and normalized for growth against the OD_600_. This experiment was repeated three times independently.

## 5. Conclusions

In conclusion, our findings indicate that the AcrAB-mediated efflux pump is a critical factor for *K. pneumoniae* survival and pathogenesis causing infections in the host. Given that AcrAB is highly conserved in many bacterial pathogens, our study provides valuable insights into the mechanism that underlies successful infections.

## Data Availability

Data available in a publicly accessible repository.

## Supplementary Materials

The following supporting information can be downloaded at: https://www.mdpi.com/article/10.3390/antibiotics13121146/s1, Figure S1: Identification of genes required for resistance to crude bile in *K. pneumoniae*; Figure S2: Growth curves of wild-type, ∆*acrA* and ∆*acrB* mutants; Figure S3: Killing assay in bile salts distinct growth phase; Figure S4: Influence of the switch-loop on SDS tolerance in *K. pneumoniae*; Figure S5: *K. pneumoniae* pathogenicity in *G. mellonella* larvae; Table S1: Characteristics of the *K. pneumoniae* strains. Table S2: The minimum inhibitory concentrations of *K. pneumoniae*.
