# Supplementary material for: AcrAB Efflux Pump Plays a Crucial Role in Bile Salts Resistance and Pathogenesis of Klebsiella pneumoniae
Source: Antibiotics (Basel). 2024 Nov 29;13(12):1146. doi: 10.3390/antibiotics13121146 (PMC11672700; doi:10.3390/antibiotics13121146)
Supplement: Supplementary file 1 [file antibiotics-13-01146-s001.zip › antibiotics-3295908-supplementary.pdf]

Table S1 Characteristics of the *K. pneumoniae* strains.

Strains	MLST[a]	Year	Source[b]	Concentration[c] (%)
A2312	ST11	2014	Feces	8
A1966	ST14	2014	unknown	10
A2280	ST15	2014	LRS	10
A2282	ST15	2014	LRS	10
A2293	ST15	2014	unknown	<2
A1682	ST17	2013	Blood	8
A1838	ST17	2014	Feces	10
A1871	ST37	2013	Urine	8
A2281	ST37	2014	LRS	6
A1824	ST65	2014	Feces	10
A1863	ST86	2013	Blood	8
A1968	ST147	2014	LRS	10
A2306	ST258	2014	unknown	8
A2359	ST307	2014	LRS	10
A2368	ST307	2014	LRS	10
A1851	ST395	2014	Feces	4
A2612	ST395	2015	Feces	6
A1805	ST437	2013	unknown	6
A2263	ST685	2014	LRS	10
A1749	ST709	2013	Feces	6
A1860	ST846	2014	Blood	10
A2366	ST875	2014	Feces	10
A2449	ST1306	2014	Urine	<2
A1876	ST1318	2014	Feces	6
A1703	ST1333	2013	LRS	8
A2369	NEW	2014	AF	10
A2371	NEW	2014	LRS	10
A2373	NEW	2014	LRS	8
ATCC BAA-2146		unknown	Urine	6
ATCC BAA-1899		unknown	unknown	8

Note: [a]MLST: multi-locus sequence typing; ST: sequence type; NEW: new ST types.

[b]Clinical isolates source. AF: ascitic fluid; LRS: lower respiratory secretions; [c]Concentration (%): concentration of bile salts.

A

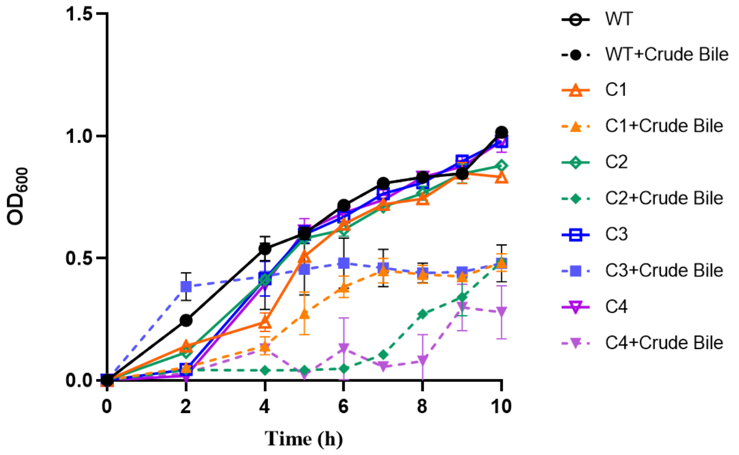

B

Strains No.	ORF	Encode protein
C1	*acrA*	Acriflavine resistance protein A
C2	*acrB*	Acriflavine resistance protein B
C3	*nplD*	novel lipoprotein
C4	*ruvB*	Holliday junction ATP-dependent DNA helicase *ruvB*

Figure S1 Identification of genes required for crude bile resistance in *K. pneumoniae*

(A) The OD_{600} of four bile salts-resistant mutants caused by transposon insertions with or without 0.5% crude bile. These cultures were then incubated with agitation at 37°C, and the OD_{600} was recorded at the specified time points. Each mutant in the transposon mutagenesis library was tested, and the wild-type A2312 was used as a control. (B) Gene locus identification of transposon insertion in four bile salts-resistant mutants. The predicted function of these genes was annotated based on BLAST analysis.

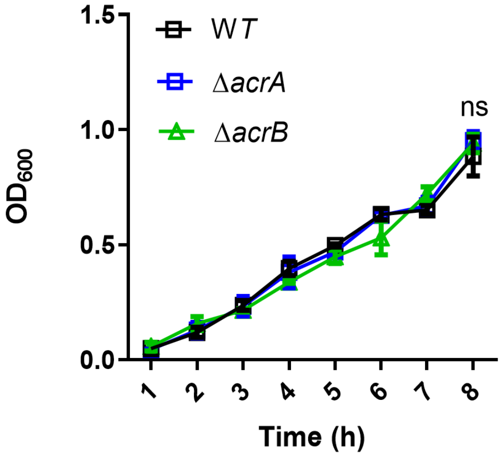

Figure S2 Growth curves of wild type, Δ*acrA* and Δ*acrB* mutants

Overnight cultures of wild type, Δ*acrA* and Δ*acrB* mutants were transferred into fresh LB medium at a 1:100 ratio and incubated at 37°C aerobically. The OD_{600} was recorded at the specific time point. The presented data represent the means ± SDs of results from three independent experiments. In cases where no statistical significance was observed, it is denoted as "ns" (Student *t*-test).

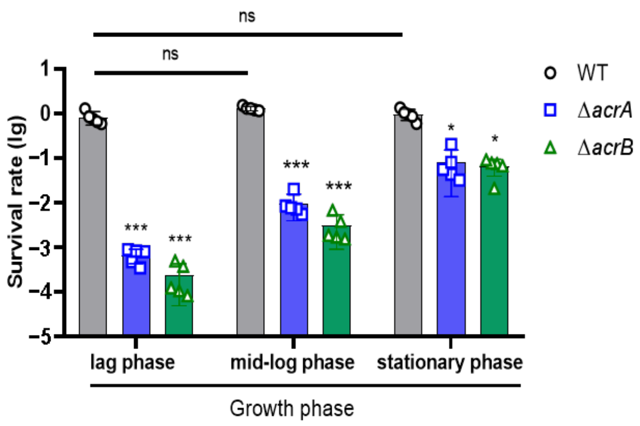

Figure S3 Killing assay in bile salts at distinct growth stages

The wild type and mutant strains were cultured in LB medium until they reached the lag, mid-log, and stationary phases. Subsequently, these cultures were diluted into both saline and saline containing 0.5% crude bile. After incubating for 1 hour, the viable cells were quantified by performing serial dilutions followed by plating on LB agar plates. The survival rate was calculated by normalizing the CFU to the group treated with crude bile. The data are expressed as means ± SDs based on the results of three independent experiments. Statistical significance is represented as follows: *, $P < 0.05$; ***, $P < 0.001$ (Student *t*-test)

Table S2 The minimum inhibitory concentrations for *K. pneumoniae*.

Antibiotic agents	MICs(μg/mL)					
	**WT**	**Δ*acrA***	**Δ*acrA*^{C}**	**Δ*acrB***	**Δ*acrB*^{C}**	**Δ*acrB*(pLG-1)**
Ampicillin	>350	>350	>350	>350	>350	>350
Streptomycin	>350	>350	>350	>350	>350	>350
Chloramphenicol	>900	200	600	600	600-900	600
Tetracycline	>480	240	>480	480	>480	240-480
Rifampicin	>20	15	20	6	20	15

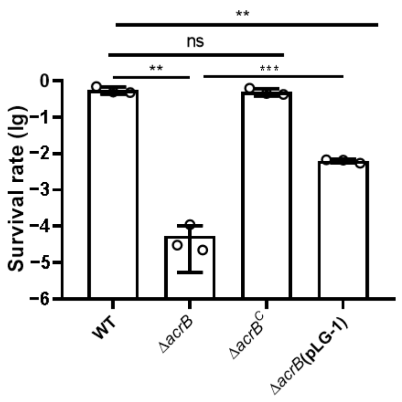

Figure S4 Influence of the switch-loop on SDS tolerance in *K. pneumoniae*

Stationary cultures of the wild type, Δ*acrB* mutant, complemented strain Δ*acrB*^{C}, and a unique complemented strain Δ*acrB*(pLG-1) with residues G615, F616, A617, and G618 deleted were diluted into saline and saline containing 0.5% SDS. After a 1-hour incubation, viable cells were enumerated. The survival rate was calculated by normalizing the CFU to the SDS-treated group. The data represent the means ± SDs of three independent experiments. **, $P < 0.01$, ***, $P < 0.001$, ns, no statistical significance (Student *t*-test).

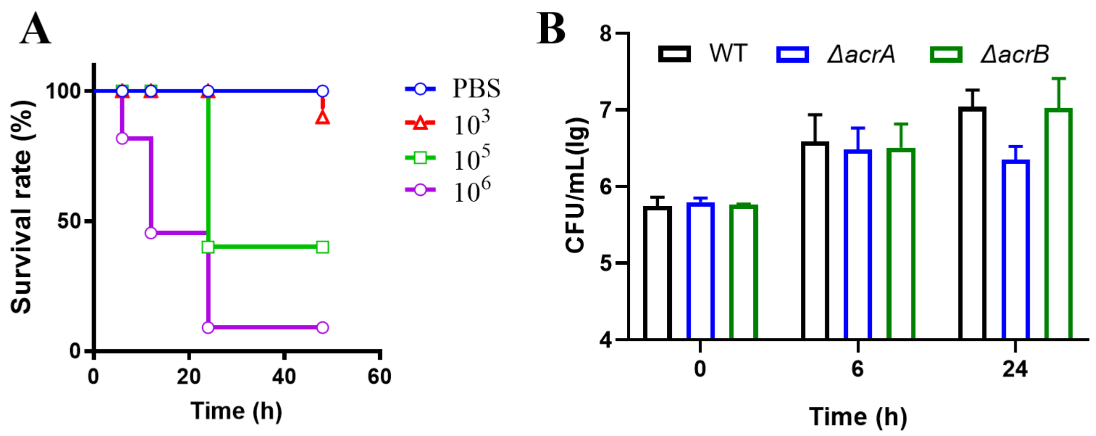

Figure S5 ***K. pneumoniae*** **pathogenicity in** ***G. mellonella*** **larvae**

(A) Determination of the lethal dose of *K. pneumoniae* in *G. mellonella*. To ascertain the lethal dose of *K. pneumoniae* A2312, various concentrations, including 10^5, 10^7, and 10^8 CFU/ml, were prepared via 10-fold serial dilutions in PBS and subsequently injected into *G. mellonella* larvae. The larvae were then incubated at 37°C in darkness, and the survival rate was assessed at specified time points (*n*=10 larvae per group).

(B) The persistence of *K. pneumoniae* in *G. mellonella* larvae. Larvae were injected with 10 μL of PBS containing a lethal dose of approximately 10^7 CFU of *K. pneumoniae*. At designated time points, hemolymph samples were collected from three individual larvae, serially diluted, and plated for quantification of bacterial CFUs (*n*=3 larvae per group).
